# Progressive research collaborations and the limits of soft power

**DOI:** 10.1007/s40037-019-0496-3

**Published:** 2019-01-28

**Authors:** Olga Kits, Camille Angus, Anna MacLeod, Jonathan Tummons

**Affiliations:** 1 Research Methods Unit, Nova Scotia Health Authority, Halifax, Nova Scotia Canada; 2 Department of Community Health & Epidemiology, Faculty of Medicine, Dalhousie University, Halifax, Nova Scotia Canada; 3 Social Dimensions of Health Program, University of Victoria, Victoria, British Columbia Canada; 4 Institute on Aging & Lifelong Health, University of Victoria, Victoria, British Columbia Canada; 5 Division of Medical Education & Continuing Professional Development, Faculty of Medicine, Dalhousie University, Halifax, Nova Scotia Canada; 6 School of Education, Durham University, Durham, UK

**Keywords:** Responsible conduct of research, Soft power, Collaboration, Research practice

## Abstract

Collaboration in diverse teams is a central topic area in medical education, health research, and healthcare. As medical education researchers we implemented an internal grant policy to develop a progressive research partnership based on widely accepted guidelines for responsible conduct of research. Our intention was to proactively manage and guide group expectations around issues such as access to data and authorship. Our policy was based on ‘soft power’ principles, using the persuasiveness of ideas, relationships and inducements to encourage people to ‘want what you want.’ This article shares how we developed and implemented the policy, experienced first-hand the limits of soft power, and it explicates some of the lessons learned.

## The story

The challenges of progressive and collaborative research practices are well documented [1–7]. As members of research teams in a variety of roles and capacities, ranging from graduate student research assistant to principal investigator, we have experienced the challenges of research collaborations. For example, we knew what it felt like to be fully involved and invested in a project only to have our contributions reduced to a line in an acknowledgement. Likewise, we knew the frustrations of limited and hierarchical access to data that inhibited full use of data and discouraged full-team collaborations. When we obtained a significant national grant in 2012 to conduct research on the technologically mediated delivery of the medical education curriculum to geographically separate campuses (distributed medical education), we purposively committed to operate our research project in a different, more progressive way.

Our research team was large, 18 people in total, each of whom had become involved with the research for their own reasons, and each of whom brought unique ideas, perspectives, expectations, and skill-sets to the table. Team members came from medical education, education, medical sociology, social anthropology, medicine, law, information technology, nursing, medical informatics, and evaluation disciplines, representing a wide range of research traditions and expectations. Our team was diverse in terms of academic and administrative responsibilities, multi-disciplinary, multi-institutional, geographically dispersed, international (Canada and the UK), largely self-organizing, and self-determining.

While we all came together over a shared interest in investigating technologies that facilitate the delivery of medical education to geographically separate campuses, many competing priorities and value systems can exist within such an interdisciplinary research team. The team demonstrated an extensive set of skills and included people who had only minimal experience with research as well as those with international reputations for cutting edge work. Relatedly, team members came to the grant with their own sets of beliefs about ‘how things are to be done’, such as expectations related to their role, level of contributions in data collection or analysis, access to data, and considerations of authorship.

With all of these factors in mind, we began our collaboration by asking the question ‘How can we work together within a publicly funded research grant to encourage a responsible, rigorous, productive and positive research environment?’ In an attempt to manage and harmonize expectations across this multidisciplinary environment, circumvent discord, level inappropriate research team hierarchy, and maximize collaboration and productivity, the local core team, without initial input from the larger team, drafted an internal grant-specific ‘Open Access to Grant Data’ policy to govern our work. The cornerstone of this policy conceptualized grant data as a common good, coupled with the following five key elements: 1) transparency and openness; 2) productive stewardship of data; 3) judicious use of public funds; 4) equitable access to grant data; and 5) fair authorship allocation. Because the funding came from the Social Sciences and Humanities Research Council of Canada, we ensured that our principles were also in alignment with Canada’s *Tri-Agency Framework: Responsible Conduct of Research*, which has as its goal the promotion of high-quality research, fairness in the conduct of research, and judicious use of public funds [8]. None of these elements are controversial in and of themselves but they are infrequently articulated or implemented as core operating principles at the start of a grant.

Borrowing a term from the international relations literature, we loosely used a strategy called ‘soft power,’ attempting to attract team members into accepting the preferences set out by our policy [9, 10]. Referencing Canadian national oversight bodies, we articulated our five policy elements as anchors of legitimacy. The idea was that rather than using hard power (such as coercion), a strategy that largely does not apply nor work in the academic world, soft power may convince people, using the persuasiveness of ideas, relationships and inducements. Our vision was for a non-hierarchical, equal-opportunity team of supportive, fully-engaged peers collaborating to contribute their different perspectives and expertise to novel and useful ways of examining our research questions. A mainstay of our vision was a team that operated and published according to ethical and equitable authorship practices.

Our assumption was that the proactive development and implementation of a transparent policy, with full buy-in, support and participation from the principal investigator, would provide explicit guideposts to members of the team, encouraging less friction, more cohesion, and supporting research accomplishments. Our soft power approach also included research-related inducements such as monies for relevant software purchases, conference travel presentations, and journal publication fees. We assumed we had been comprehensive and considered every possible outcome in the development of the policy which specifically articulated how members could, for example, take active steps towards starting a sub-project, such as writing a paper based on shared grant data. We anticipated that team members would be excited about the possibility of participating in research that provided more equal opportunities for everyone, rather than function in more traditional hierarchical ways. We were certainly surprised that the policy did not play the role or produce the outcomes we expected.

## Surprising outcomes

After developing the policy, we began the process of introducing and implementing it. The small team who developed the policy were proud of it, and of the philosophies underlying it, so we looked forward to an ongoing conversation, hoping that team members would see it as an opportunity to explore the variety of ways in which they might participate in and benefit from the research. We circulated drafts by email and gave the policy a place as a standing agenda item at research team meetings. Yet, each time the policy agenda item arose, the conversation was minimal.

The discussions that did take place seemed to unilaterally view the policy in terms of ‘generosity.’ As examples, when we highlighted that we would support any and all team member(s) to take the lead on writing a paper using grant data, the response was ‘that’s very generous.’ Likewise, when we highlighted a process whereby team members could request ‘chunks’ of data to explore sub-questions related to the larger research project, the team commended our ‘generosity.’ This was surprising, since we had initiated the policy hoping to inspire knowledge creation and unfettered collaboration and had not conceptualized it in terms of ‘generosity.’

The notion of generosity is particularly interesting and speaks to a sense of individual ‘ownership’ with respect to data. Our funder for this project, the Social Sciences and Humanities Research Council of Canada, operates under the principle that the advancement of knowledge in the social sciences and humanities is facilitated by researchers sharing data because ‘[s]haring data strengthens our collective capacity to meet scholarly standards of openness by providing opportunities to further analyze, replicate, verify and refine research findings. Such opportunities enhance progress within fields of research, avoid duplication of primary collection of data, as well as support the expansion of interdisciplinary research’ [11]. There is a traditional notion that the principal investigator is the owner of the data, and that ‘sharing’ data is a generous act. This reflects deeply entrenched ideas about research hierarchy, based in competitiveness and possessiveness. The alternative is that data sharing supports scientific advancement, transparency, and innovative knowledge production for the common good.

While no one openly disagreed with the policy, as the project progressed, more conventional research practices began to emerge. For example, with respect to authoring papers, our policy required sharing manuscript ideas with the team up front in the form of a proposal. Those who were interested in being involved were then expected to ‘sign-up’ to contribute to the writing. Per generally-accepted authorship criteria within biomedical sciences [12], co-authors had to be willing to actively put pen to paper (rather than edit an already drafted contribution) to be named as an author. When we shared our first manuscript proposal with the team, almost everyone ‘signed-up’ to be a co-author. When we then asked team members which piece of the manuscript they wanted to write, and to identify a timeline by which the section would be completed, more than half of the original potential authors declined to participate.

The policy also argued for a flattened grant hierarchy and equitable access to grant data for all research team members, from research assistants to co-applicants. Our multidisciplinary group, we reasoned, would have unique ways of exploring, understanding and analyzing data. And given that it was a publicly funded grant, we wanted to facilitate productive stewardship of the data accordingly. The policy opened the possibility, with financial and other supports in place, for any team or staff member to be an initiator or owner of a sub-project within the grant, perhaps bringing an interesting idea to fruition to present at a conference or in an eventual manuscript. Contrary to our expectations, this concept had almost no uptake.

It became clear to us that typical, hierarchical ideas about how research collaborations work are deep-rooted. Likewise, simply putting our blended model of soft power and incentives into text and labelling it a policy, holding ongoing discussions about the policy as a group, and setting expectations, was not enough to modify these strongly held beliefs and behaviours. And, despite these efforts to encourage broad participation for the larger team, a key, and much smaller, group of actively engaged researchers emerged, and were responsible for producing most of the scholarship outcomes related to the grant.

For those few team members who were able to work in general agreement with its principles, our resultant collaboration was meaningful and productive. We published in more journals and presented at a wider range of conferences than those that were traditionally on our radars, thus increasing the impact of sharing our research findings. Perhaps more importantly, we discovered and established a new cohort of similarly inclined colleagues with whom we can continue to engage in high quality, collaborative ethical medical education research.

## Lessons learned

We had hoped that implementing a soft power policy would lead all or most members to want what we wanted: a project philosophy that valued joint ownership, collaboration, investment and innovative knowledge production. We naively assumed that a pro-active approach and codified ground-floor policy based on current best practices and ongoing engagement with its principles would lead to a more engaged, responsible, ethical, cohesive, fair and productive grant experience. While in conversation, research team members thought that introducing a policy designed to align ideals of responsible conduct of research with self-interest was attractive, in practice it turned out to be much more challenging. In addition to the positive features we have described, we also encountered challenges related to our overall approach and policy, which we present here as considerations and lessons learned.

### Characteristics of temporary research groupings

Changing the ways in which we engage in collaborative research involves challenging deeply held expectations and traditions about what it means to be a member of a research team. However, academic research collaborations also occur in particular contexts and tend to exhibit certain characteristics that can both facilitate and constrain: high diversity, geographically dispersed, technologically bounded, voluntary, and self-organizing. In addition, this formation was only temporary (3 years) and lived within a porous ecosystem, where members’ main affiliations were elsewhere as well as multiple. Therefore, members had the option to engage as much or as little as they wished, and our ability to attract and persuade had limits. We now recognize that within the constraints described there are limits to meaningful engagement on complex and sensitive matters.

### Be inclusive and consultative when designing and implementing the policy

Despite our intention to design a policy to encourage collaboration and shared participation in authorship, the policy itself was initially developed by a small group of researchers who were aware of current ethical conduct of research expectations, and committed to its principles. In spite of multiple attempts to discuss the policy as the team was initially developing, we believe we may have inadvertently created a context in which other members of the research team felt excluded or unprepared to operate within this new (to them) paradigm. Also, some members of the research team found the policy difficult to conceptualize and apply. This may have been based on the fact that the model of collaboration espoused by the policy was quite different than the standard ‘way of doing business’ in a traditional academic institutional context.

### Build appropriate timelines and ensure project completion

Any policy is held in place by a set of related processes. In the case of our ‘Open Access to Grant Data’ policy, these processes included proposing a topic, inviting participation, scheduling meetings, integrating multiple authors’ contributions, and sharing drafts. The policy, through its processes, may have contributed to a sense of decreased efficiency and increased administration. Our policy also allowed for individual researchers to propose an idea for a contribution (paper, presentation, etc.). Once proposed, it was the responsibility of the proposer to steer the contribution to completion. A challenge with this approach is the relative ease of proposing a contribution as opposed to actually engaging in, and completing, the proposed work. We learned that a risk with this approach is that good ideas, once claimed by a member of the research team, could become lost, held hostage, or never find their way to completion.

### Ensure team members are comfortable with technological tools

Our policy was backed by a set of technological resources designed to maintain transparency and accountability, and enable access for all geographically dispersed team members. We conceptualized this as a reasonable, fair and equitable way for team members to access grant information; however, we had not considered the wide variety of technological skill and comfort amongst our team members. The technological solutions we employed were efficient and served our purpose well; however, for some, they acted as a barrier. This could be seen as ironic given that our project was investigating technologies that facilitate distance learning.

## Moral of the story

We learned that proactively applying a blended model of soft power and incentives had its limits in achieving our broader goals of developing a progressive and collaborative research partnership. While some of the open access policy elements helped create traction for cooperation, there was scant engagement with the broader aspirational policy ideas. Technology and administrative processes intended to facilitate participation acted as barriers. Additionally, the dispersed and temporary nature of the team set limits on the extent to which people could be persuaded to adopt a new research culture. Some of these obstacles could have been addressed with greater inclusion in grant policy development.
